# Education Research: Making a Tweetorial Fly: Features of Educational Social Media Posts Associated With High Sharing and Engagement

**DOI:** 10.1212/NE9.0000000000200160

**Published:** 2024-10-31

**Authors:** Catherine S.W. Albin, Tianwen Ma, Gabriela F. Pucci, Aaron S. Zelikovich, Eric C. Lawson, Neil Dhruva, Simone Masiero, Aarti Sarwal, Neha S. Dangayach, Aaron L. Berkowitz, Nicholas A. Morris, Lyell K. Jones

**Affiliations:** From the Departments of Neurology and Neurosurgery (C.S.W.A., E.C.L.), Emory University School of Medicine, Atlanta, GA; Division of Biostatistics (T.M.), Rollins School of Public Health, Emory University, Atlanta, GA; Department of Neurology (G.F.P.), University of Pittsburgh, PA; Department of Neurology (A.S.Z.), Weill Cornell Medical College, New York, NY; Emory University School of Medicine (N.D.), Atlanta, GA; Consulting Web Developer (S.M.), Scotland; Department of Neurology (A.S.), Wake Forest University, Winston-Salem, NC; Departments of Neurology and Neurosurgery (N.S.D), Icahn School of Medicine at Mount Sinai, New York, NY; Department of Neurology (A.L.B.), University of California, San Francisco; Department of Neurology (N.A.M.), University of Maryland School of Medicine, Baltimore, MD; and Department of Neurology (L.K.J.), Mayo Clinic, Rochester, MN.

## Abstract

**Background and Objectives:**

Social media platforms such as X (formerly Twitter) are increasingly used in medical education. Characteristics of tweetorials (threaded teaching posts) associated with higher degrees of engagement are unknown. We sought to understand features of neurology-themed tweetorials associated with high sharing and engagement.

**Methods:**

We created a neurology-themed tweetorials data set by searching “tweetorial” AND “neurology” on X that were posted between November 2018 and December 2022. Tweetorial and author characteristics were collected from X and by viewing the author's academic profile. We created and validated a novel formula to determine the tweetorial's “X Factor” (XF), a measure of reader engagement and distribution, reflecting reposts and likes. Each tweetorial was analyzed for basic variables, the author's academic rank, and thematic content. Each first post underwent a language analysis using Linguistic Inquiry and Word Count (LIWC-22) and was hand-coded for style (such as “statement” or “clinical case”). We determined each covariate's impact on XF. The general estimating equation was applied to correct for the author effect.

**Results:**

We identified 392 neurology-themed tweetorials posted by 96 unique authors. XF strongly correlated with impressions (*R*^2^ = 0.85) and was validated in a separate data set (*R*^2^ = 0.74). The median XF of the tweetorials was 28.5K (interquartile range 12.7K–61.5K). Tweetorials about a “General Neurology Topic” and with a “Clearly Stated Topic” had 48% and 49% higher XF than those without (*p* = 0.001 and 0.006, 95% CI 17%–88%, 12%–97%, respectively). Having a “creative” first post, including a unique hashtag, and featuring an author-made graphic correlated with 60%, 49%, and 84% higher XF than posts without those elements (*p* = 0.01,95% CI 13%–125%, *p* < 0.001, 95% CI 16%–92%, *p* < 0.001, 95% CI 30%–164%, respectively). Continuing medical education (CME) accreditation and higher scores on “positive tone” negatively affected XF (−80%, *p* < 0.001, 95% CI 70%–86% and −7%/point of positivity, *p* < 0.001, 95% CI 2%–10%, respectively).

**Discussion:**

Tweetorial engagement and distribution are determined by multiple factors including authorship, clarity of the topic, and visual appeal of the post. CME accreditation was strongly negatively associated with sharing and may reflect a sharing preference for personal accounts over institutional ones, although further study is needed.

eLearning, education that is provided through a digital platform, is an increasingly recognized teaching paradigm in medical education and neurology. A recent scoping review of medical education innovations found that eLearning educational innovations have been the second most frequently published type of education innovation in neurology.^1^ The scope of eLearning is broad, but of the many forms, social media–based teaching through X (formerly Twitter) has several advantages: X is widely used by the medical community, is accessible globally, and is free to use. However, before 2018, all teaching needed to be delivered within the character limit of a single (280-character) post. In 2018, X enabled “threads” of posts—series of posts that could be linked together. As a result, the tweetorial—a portmanteau of “tweet” and “tutorial”—emerged, which is a series of posts with an educational aim. Tweetorials are well adapted to adult learner preferences, being delivered asynchronously, with distilled points, and take a short investment of time to read.^2^ In addition, they can reach a large number of X users without any associated costs.

X-based teaching in neurology has increased in recent years. A neurocritical care educational account engaged learners from 22 different countries and multiple subspecialties with tweetorials on neurologic emergencies.^3^ An initiative called #NeuroPostitPearls (tweets with brief pearls about neurologic emergencies) reached users in 58 countries.^4^ Although online resources are perceived by students as important in learning neurology/neuroscience,^5^ studies on teaching through social media have shown that readers' engagement with neurology content fluctuates or wanes.^3^ To be widely shared, teaching delivered through X must attract users' attention and be “liked” or “reposted.” This endorsement signals the X algorithm to place the post on the “Timelines” of other similar social media users, even if they are not following the author.^6^ Therefore, delivering a message that is “like-able” and “repost-able” is the key first step in reaching a broad audience. This phenomenon highlights a distinguishing feature of using social media as an education platform: the ultimate impact of the post is directly influenced by the engagement of the learners.

In this study, we sought to describe and validate a social media impact factor that would account for “reposts” and “likes,” reflecting engagement and sharing. We have termed this the “X Factor” (XF). Subsequently, we sought to determine which features of neurology-themed educational social media posts best correlated with high impact, as defined by a high XF.

## Methods

### Identification of Tweetorials

Using the search bar in X, we first searched (“tweetorial” AND “neurology” OR “neuro”). We visited the profile of any X user who was associated with this search and then specifically searched their profile for the 2 keywords “tweetorial” and “thread.” We also reviewed the author's profile for any “Moments” (a disabled feature of X, which allowed users to make a collection of their tweetorials) and included any neurology-themed tweetorials within these “Moments.”

If any author used a specific hashtag (a word or phrase preceded by # that marks the content topic on social media, such as #EndNeurophobia or #ContinuumCases) for their tweetorials, we separately searched for this hashtag to collect any associated tweetorials.

We then screened an online, independently maintained, open-access tweetorial database, medtweetorials.com, for all neurology-themed tweetorials and included any unique tweetorials not already been captured through the search methods given above.

### Creation of the XF

To understand what makes some tweetorials more widely shared than others, we first had to define a measure of success that could be described using publicly available metrics, which would facilitate comparison between tweetorials that were authored over the several years. A tweetorial's “impression” count is the total number of times a post is seen by any global user through their time line or search result.^7^ While this is now publicly listed below the post, until December 2022, this was only known to the post's author. “Impressions” is an excellent marker of dissemination, given it is a direct measure of distribution. However, because this was not publicly known for all tweetorials, we developed an equation using “likes,” “reposts,” and “quote reposts,” which are markers of impact and engagement, and, most importantly, metrics that have always been publicly listed. Using the “impressions,” “likes,” and “repost” data from 34 tweetorials authored by members of the study group before December 2022, we designed a formula to approximate “impressions” (in thousands) from the publicly available information. We termed this the “X Factor” (XF) of a tweetorial, which is directly influenced by features of engagement (likes) and sharing (reposts and quote reposts):

$$X\;\text{factor}=\sqrt{\text{Likes}}+\frac{\text{Quote}+\text{Reposts}}{2}\times 1,000$$

**Figure 1:**
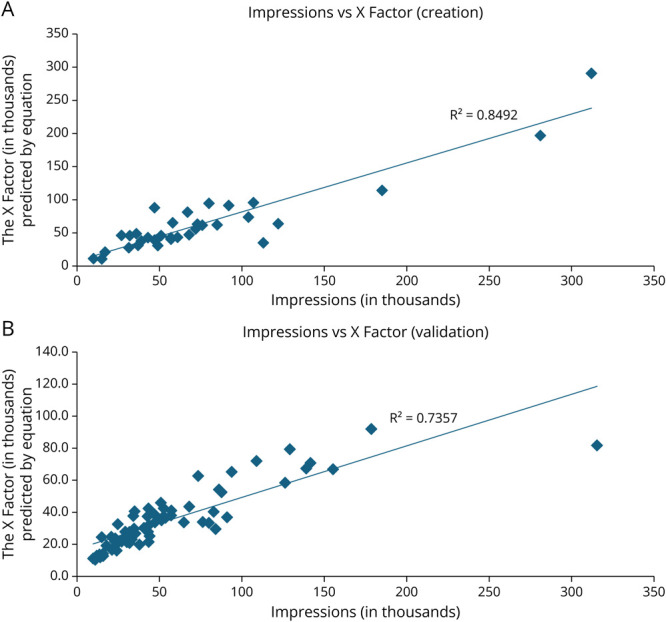
Creation and Validation of the Formula for a Post's X Factor (A) Study authors authored 34 tweetorials that were written before December 2022. These impressions were not publicly available but known to the author and shared with our team. Impressions were correlated against the sharing predicted by the X Factor equation. (B) Validation of the impact factor on the #ContinuumCases data set. The impact factor was validated in a data set of 72 #ContinuumCase tweetorials, which were mostly authored after collection of the tweetorial data set analyzed in this article was complete. 7 tweetorials were duplicates and removed.

This equation was based on the observation that “quote reposts” and “reposts” were more likely than “likes” to signal to the X algorithm (which is not publicly available) that the user found this type of content engaging. “Reposts” and “quote reposts” also place the tweetorial on another user's time line and thus increase the tweetorial's visibility while “likes” do not. The XF formula was tested using a regression analysis of collected “impressions” vs the derived XF. This comparison resulted in an *R*^2^ of 0.85 (Figure 1A). This formula was validated in a separate tweetorial data set, which confirmed excellent correlation (Figure 1B). This equation is specific to posts on X and a different formula than what has previously been described to calculate a scientific journal's X Impact Factor.^8^

### Basic X Variables

For each tweetorial, we collected basic data including the author's account handle (name of the account, which is preceded by @); the date and time posted; and the number of likes, retweets, and quote retweets. We also collected the total number of hashtags, the number of graphics interchange formats (GIFs, animated images), and the number of other X users mentioned within the thread. To assess the degree of linkage with neurology literature, we captured the number of PubMed links in each tweetorial and the total number of links.

### Author Variables

To understand the impact of the author, we visited each X handle's “bio” as listed on their X profile and collected the date the author joined X, their following at the time of data collection, and the total number of tweets at the time of data collection. We also included the author's specialty and academic position. If specialty and rank were not listed in the X profile page, we used Google to find their academic profile. We also included verified status (at the time of data collection, which included “legacy”/notable status and paid membership). Finally, we documented whether the author had a tweetorial collection (i.e., an X “Moment”) or whether the tweetorial being analyzed was a “pinned tweet.”

### Thematic Analysis of the Tweetorial

We conducted a thematic analysis by categorizing the content and theme of each tweetorial regarding the subspecialty (general neurology, neurocritical care, vascular neurology, etc.) and the target audience to likely benefit from it. Tweetorials were scored as a “General Neurology Topic” if they were about the practice of neurology, neuroanatomy, neurobiology, medical history, neuroradiology, or professional development. Tweets that were about a specific branch of neurology (vascular neurology, neuroimmunology, pediatric neurology, etc.) were coded as “Specialized Topic.” Examples are shown in eFigure 1.

Based on the cumulative impression of the entire tweetorial, tweetorials were coded as “Of Broad Interest” if they had perceived utility to a medical student or a non-neurology physician audience. Tweetorials that were likely only to be of interest to neurologists (residents, fellows, and attendings; beyond the scope of what a medical student would be reasonably expected to know) were scored “Of Narrow Interest” (examples in eFigure 2). Note that, though similar, “General Neurology Topics” were not necessarily “Of Broad Interest.” For example, a tweetorial about positive physical examination findings and the sensitivity for diagnosis of functional neurologic disorder (FND) was scored as “General Neurology Topic,” given the prevalence of FND in many general and subspecialty clinics; however, because the focus of the tweetorial was on the sensitivities of various examination maneuvers for ruling in FND, this tweetorial was scored as “Of Narrow Interest,” given that it was a high-level discussion that would likely be beyond the interest of a non-neurologist.^9^

If the first post made clear what would be discussed in the tweetorial, the tweetorial would be scored as “Clearly Stated Topic.” If it did not, then it would be scored “Topic Not Revealed.” Examples are shown in eFigure 3.

To assess the authors' ability to demonstrate best teaching practices as articulated by Bloom et al. where the cognitive domain of teaching is organized into clearly stated learning objectives,^10^ we assessed how often learning objectives were clearly stated in the tweetorials. To assess the validity of the content, we screened each tweetorial for whether the tweetorial was peer-reviewed or was accredited for continuing medical education (CME).

### First Tweet Analysis

The first tweet is the most visible of the tweetorial and is displayed on followers' “Timeline” (a user's homepage that contains posts from users they follow or those that the X algorithm predicts would be of interest to the user). As such, this first post often determines whether the tweetorial is of interest to the reader. We assigned a designation of “statement,” “clinical case,” “creative,” “humorous,” or “question” to each first post (examples of each type in eFigure 4). First tweets were scored as “creative” if they featured current event, poem, game, puzzle, or a direct quote. “Humorous” tweets were lighthearted. “Clinical case” was assigned if the first tweet was about a case. “Question” was scored when the focus of the first tweet was to ask a question. “Statement” was scored if the first tweet was a statement about what would follow or a fact.

We assessed the visual appeal of the first tweet by assessing the use of hashtags, emojis, and mentions. Hashtags and mentions appear blue and thus provide visual interest to the post. We also coded whether the first post included a image or GIF and whether the figure was an image likely to have been made by the author (example in eFigure 4, A and B). We specifically coded whether a “unique hashtag” was used. We defined “unique hashtag” as a recurring hashtag that was used deliberately by the author to signal their teaching cases. For example, cases from Continuum use the hashtag #ContinuumCases; teaching cases from the World Stroke Academy (@WorldStrokeEd) use #WeekendLecture. An author's unique hashtag might then be adopted by other authors. For example, #EndNeurophobia (created by @AaronLBerkowitz) became widely used by others posting teaching cases on X.

### Language Analysis

Each first tweet underwent a language analysis using Linguistic Inquiry and Word Count (LIWC-22) software with their traditional dictionary, which coded the first post in each tweetorial language as part of 9 variables: I-words, positive tones, negative tones, social words, cognitive processes, allure, moralization, analytic, and authentic.^11^ Each variable is graded on a scale of 0–100, 0 meaning the text scores very low on that variable and 100 being the highest possible.

### Interrater Reliability for Subjective Elements

Several elements of the tweetorial scoring were subjective (i.e., what counted as a “General Neurology Topic,” what was “Of Broad Interest,” what was a “Clearly Stated Topic,” and the style of the first tweet). To improve standardization, 1 scorer (C.S.W.A.) scored each of these subjective categories. To test the interrater reliability of scoring, 100 randomly selected tweetorials were then sent to a second attending neurologist (N.A.M.) who scored each of these 100 tweetorials as either “General Neurology Topic vs Specialized,” “Of Broad Interest vs Of Narrow Interest,” or “Clearly Stated Topic vs Topic Not Revealed.” N.A.M. also evaluated the style of the first tweet. Because there was only “fair” agreement of what would be “Of Broad Interest” between the 2 attending neurologists, a medical student (N.D.) was recruited to score all 392 tweetorials on this topic as the consensus of the study group was that as a non-neurologist and student, this rater would provide the highest validity for topics that are of interest and utility to the non-neurologist population. The student's score was used for this covariate in the final model to derive its impact on XF.

### Statistical Methods

The complete statistical methodology is given in eMethods.

### Standard Protocols

The Institutional Review Board at Emory University deemed this research “exempt” because it did not meet the definition of “human subject research” or “clinical investigation.”

### Data Availability

Data were not provided in the article because of space limitations, but they may be shared (anonymized) at the request of any qualified investigator for purposes of replicating procedures and results.

## Results

### Identification of Tweetorials

We identified 392 neurology-related tweetorials posted between November 2018 and December 2022. These tweetorials were authored by 88 accounts and 96 authors (because some accounts allow multiple authors to write tweets from that account).

The median number of “likes” for the first tweet of the thread was 101 (interquartile range [IQR] 37.25–274.75). The median retweet number and quote retweet number were 30.5 and 5, respectively (IQR 11–76.7 and 2–11, respectively). The median XF among the tweetorials was 28.5K (IQR 12.7K–61.6K).

### Basic Variables

Table 1 provides basic variables for the tweetorials. Tweetorials contained a median of 5 hashtags (IQR 2–10) and 3 mentions (IQR 0–10). 29% (114/392) of tweetorials contained a PubMed link, and 27.8% (109/392) contained a poll.

**Table 1:** Descriptive Statistics of Tweetorial Variables

**Basic Variables: Binary**		
Time posted	am (n = 113, 29%)	pm (n = 279, 72%)
Day	Weekday (n = 296, 76%)	Weekend (n = 96, 24%)
PubMed link	Yes (n = 114, 29%)	No (n = 278, 71%)
Poll	Yes (n = 109, 28%)	No (n = 283, 72%)

**Basic variables: continuous variables**	**Median**	**Interquartile range**
Tweetorial length	13	9–19
Log (tweet length)	2.57	2.2–2.94
GIF count	0	0–1
Hashtag counts	5	2–10
Mentioned counts	3	0–10
PubMed link count	0	0–1
Total link count	2	0–5

**Content and theme: binary variables**	**Yes (n, %)**	**No (n, %)**
“General Neurology Topic”	167, 43%	225, 57%
“Of Broad Interest” scored by N.D.	225, 58%	161, 42%
“Clearly Stated Topic”	312, 80%	80, 20%
Learning objectives clearly stated	58, 15%	334, 85%
Accredited for CME	18, 5%	374, 95%
Mentions peer review	88, 24%	304, 76%

**First tweet features**	**Yes**	**No**
Statement	199	193
Creative (poll, current event, quote retweet, etc.)	41	351
Humorous	19	373
Used a unique hashtag	103	289
Used emoji	208	184
Used hashtags	328	64
Mentioned other users	191	201
Author-made graphic	67	325
Any graphic	269	123

**Language analysis: continuous variables**	**Median**	**Interquartile range**
I-word	0	0–2.6
Positive tones	2.2	0–3.6
Negative tones	0	0–2.0
Social words	2.8	0–5.4
Cognitive processes	6.7	2.9–11.6
Allure	3.6	0–6.4
Moralization	0	0–0
Analytic	89.5	71.2–96.7
Authentic	16.7	4.1–52.2

Abbreviations: CME = continuing medical education; GIF = graphics interchange format.

The denominator of “Of Broad Interest” is 386 because 1 Twitter user's account had been deactivated at the time of N.D.'s scoring.

The general estimating equation (GEE) model for basic variables (Table 2) demonstrated that, after accounting for authorship status, there were no significant difference in impact based on the time of day posted, whether the tweet was posted on a weekend or weekday, or whether the tweetorial included PubMed links. Including a poll resulted in 73% less XF (*p* < 0.001, 95% CI −28 to −131%) while each additional link resulted in 4% less XF (*p* = 0.02, 95% CI −1% to −8%). Tweetorial length was skewed with several very long outliers. After a logarithmic transformation, log (tweetorial length) was significantly positively associated with impact; longer log(tweetorial) had 63% more XF (*p* < 0.001, 95% CI 31%–103%).

**Table 2:** GEE Model Results of Basic Covariates (QIC = 408.2)

Covariates	Estimate	95% CI low	95% CI upper	*p* Value
am vs pm	0.15	−0.09	0.38	0.22
Weekend vs weekday	0.14	−0.14	0.42	0.32
Log (tweetorial length)	0.49	0.27	0.71	<0.001
GIF presence	−0.12	−0.41	0.18	0.43
Presence of hashtags	−0.36	−0.90	0.18	0.19
Presence of mentions	−0.27	−0.66	0.11	0.17
Presence of a PubMed link	0.22	−0.10	0.53	0.17
Total number of links (continuous variable)	−0.04	−0.08	−0.01	0.02
Presence of a poll	−0.55	−0.84	−0.25	<0.001

Abbreviations: GEE = general estimating equation; GIF = graphics interchange format; QIC = quasi-likelihood under the independence model criterion.

### Author Variables

Authors were stratified by specialty and academic rank as shown in eFigure 5, A and B. Adult neurologists were the most frequent author type (61.5%, 59/96). Assistant professors were the most frequent academic rank (22.9%, 22/96). Some authors (15.9%, 14/88) used the “Moment” feature to collect their tweetorials. 5 accounts (5.6%) were verified; however, only 1 was verified under the “legacy” standard (meaning the user had been vetted by X as a noteworthy and verified source, in contrast to the current system in which paying users have a “blue checkmark”).

The GEE model for author variables (Table 3) showed that, after accounting for authorship status, non-neurology authorship correlated with higher impact (*p* < 0.001). Tweets authored by a neurologist resulted in 48% decrease in XF compared with those by non-neurologists (*p* < 0.001, 95% CI 19%–67%), adjusting for other covariates. Authors with “Moments” and “pinned tweets” were more impactful than those without (*p* < 0.001 and 0.04, respectively). X users who used the “Moment” feature had posts with 82% higher in XF than those of authors who do not use this feature (*p* < 0.001, 95% CI 42%–132%), adjusting for other covariates. The XF of a post that was pinned to the author's profile was 5.0 times larger than the XF of a nonpinned post (*p* = 0.04, 95% CI 1.1–22.0), adjusting for other covariates.

**Table 3:** GEE Model Results of Author Covariates (QIC = 396.2)

Covariates	Estimate	95% CI low	95% CI upper	*p* Value
Author is a neurologist	−0.66	−1.12	−0.21	<0.001
Author is verified	−0.14	−0.81	0.53	0.68
Author has a collection of tweetorials	0.60	0.35	0.84	<0.001
Author had a “pinned” tweet	1.60	0.10	3.09	0.04

Abbreviations: GEE = general estimating equation; QIC = quasi-likelihood under the independence model criterion.

### Thematic Analysis of the Tweetorials

22% (87/392) of tweetorials were about vascular neurology. A full breakdown is shown in eFigure 6. Topics that were scored as “Clearly Stated” and as a “General Neurology Topic” were significantly associated with higher impact, with, on average, 48% and 49% higher XF than those without (*p* = 0.001 and 0.006, 95% CI 17%–88%, 12%–97%, respectively), adjusting for other covariates.

22% (88/392) of tweetorials mentioned being reviewed by another physician, 15% (58/392) had clearly stated learning objectives, and 5% (18/392) were written for CME credit. The GEE model showed that tweetorials with CME accreditation had 80% less XF than those without (*p* < 0.001, 95% CI 70%–86%), adjusting for other covariates (Tables 4

**Table 4:** GEE Model Results of Thematic Analyses (QIC = 392.2)

Covariates	Estimate	95% CI low	95% CI upper	*p* Value
Scored as “General Neurology Topic”	0.39	0.16	0.63	0.001
Scored as “Of Broad Interest”	−0.13	−0.39	0.13	0.33
Scored as “Clearly Stated Topic”	0.40	0.11	0.68	0.006
Clearly stated learning objectives	−0.04	−0.33	0.26	0.80
CME accredited	−1.61	−2.00	−1.22	<0.001
Stated the tweetorial was peer-reviewed	−0.02	−0.25	0.21	0.87

Abbreviations: CME = continuing medical education; QIC = quasi-likelihood under the independence model criterion.

6 NAs are due to the missing tweets.

**Table 5:** GEE Model Results of First Tweet Analyses (QIC = 403.9)

Covariates	Estimate	95% CI low	95% CI upper	*p* Value
First tweet style: question	0.17	−0.24	0.58	0.41
First tweet style: clinical case	−0.14	−0.49	0.20	0.42
First tweet style: creative	0.47	0.12	0.81	0.01
First tweet style: humorous	0.29	−0.31	0.90	0.34
First tweet: statement	Reference			
First tweet uses a unique hashtag	0.40	0.15	0.65	<0.001
First tweet number of hashtags	0.01	−0.04	0.06	0.66
First tweet number of mentions	−0.02	−0.04	0.01	0.16
First tweet has an author-made graphic	0.61	0.26	0.97	<0.001
First tweet has any graphic	0.09	−0.20	0.37	0.56

Abbreviations: GEE = general estimating equation; QIC = quasi-likelihood under the independence model criterion.

### First Post Analysis

51% (199/392) of first posts were statements (Table 1, eFigures 4 and 7). Using the χ^2^ test on the entire contingency table, there was no statistical significance between the style of the first tweet and its XF. However, when statement was used as a reference in the GEE model, tweetorials with “creative” first posts (eFigure 4) had 60% increase in XF than tweetorials with “statement” first posts (*p* = 0.01, 95% CI 13%–125%), adjusting for other covariates. Other first post variables associated with higher XF were “a unique hashtag” that resulted in 49% increase in XF (*p* < 0.001, 95% CI 16%–92%) and having an “author-made graphic in the first tweet” (eFigure 4, A and B), which resulted in 84% increase in XF (*p* < 0.001, 95% CI 30%–164%) (Table 5).

### Language Analysis

Table 1 presents the language analysis of the first tweet. Tweetorials generally scored low on all the variables, but highest on “cognitive processes” (median 6.74, IQR 2.94–11.55) and “analytic” (median 89.5, IQR 71.22–96.72). The GEE model in eTable 1 showed that 1-unit increase in positivity, on average, resulted in 7% decrease in XF (*p* < 0.001, 95% CI 2%–10%), adjusting for other covariates.

### Final Model With Stepwise Selection by Quasi-Likelihood Under the Independence Model Criterion

eTable 2 presents the model that best predicted tweetorial sharing as defined by the highest XF. This model analyzed the effect of all variables found to be significant within their respective domain (basic, author, theme, first post). “Non-Neurologist” as an author variable and “Author-Made Graphic” in the first post were most significantly predictive of a high XF. A significant negative impact on XF was attributed to CME accreditation.

### Interrater Reliability

There was substantial agreement in scoring the style of the first post (eTable 3). There was moderate agreement between 2 attendings about whether a topic was a “General Neurology Topic” and “Clearly Stated Topic” and fair agreement on what constituted “Of Broad Interest.”

## Discussion

When used purposefully and thoughtfully, X can provide members of the medical community with access to information, expert opinion, and diverse perspectives.^12^ In the realm of teaching through X, the tweetorial has emerged as a powerful strategy to offer a more in-depth coverage of a topic than what could be covered in a single post while still adhering to the brevity inherent to the platform.^2^ While there are guides about leveraging X in medical education^13,14^ and for creating tweetorials,^2,15^ these were based on authors' experiences, without objective support for what makes certain educational social media post successful regarding likes and sharing.

Using publicly available metrics (“likes,” “posts,” and “reposts”), we determined a score (the X Factor, XF) that closely matched the tweetorial's first post's “impressions” and could be used to compare data across multiple years, before “impressions” were publicly available. Our equation had excellent correlation and was validated in an external data set (Figure 1, A and B). A strength of this study is demonstrating the feasibility of determining impact across a large data set using metrics available on many social media platforms. Furthermore, how impressions are generated is related to the X algorithm and is subject to change; however, the XF is derived entirely from transparent metrics that have always been available to all users and are unrelated to qualitative analysis or educational intent. Thus, even when “impressions” are given, the XF is a standardized equation that is more transparent and less dependent on an algorithm. As such, it may be a more standardized way to compare a post's impact and may have applications in other social media analyses where likes and reposts are also publicly available.

**Figure 2:**
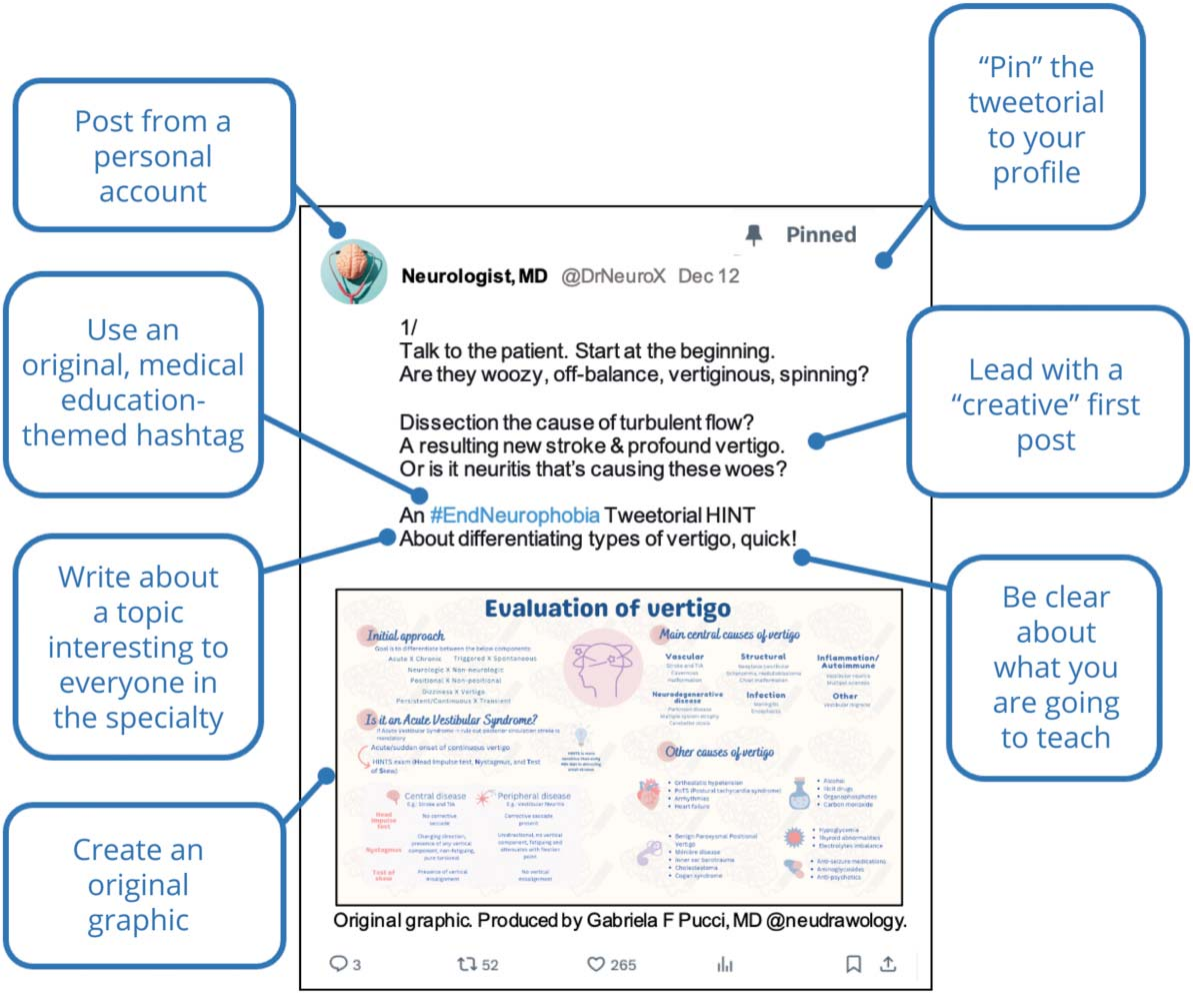
Anatomy of High X Factor First Post A visual summary of elements that are associated with a high X Factor (engagement and sharing).

Using a high XF to define success, our analysis of nearly 400 neurology-themed tweetorials demonstrated multiple features of a tweetorial that both correlate with a high XF and can be deliberately engineered by the author (Figure 2). Having an author-made graphic, a creative hook (such as a game, poem, or current event reference, eFigure 4), and a unique hashtag (such as #EndNeurophobia or #NeurologyMorningReport) in the initial post was positively and significantly correlated with sharing and engagement. This mirrors what is seen in the commercial industry where highly visual content is the most widely liked and shared.^16^

Thematically, tweetorials about a “General Neurology Topic” and those with a “Clearly Stated Topic” had higher sharing and engagement. This is perhaps unsurprising and affirms what is known about teaching in any context—having a Clearly Stated Topic and learning objectives helps frame learning.^17^ Of interest, given the assumed short attention span of a social media user, a log transformation of the length demonstrated that longer tweetorials have higher XF. However, it is worth noting that most of the tweetorials in our sample were still short (<19 posts in length, median length 13); thus, even longer tweetorials fall into X-users’ self-reported preference of reading tweetorials that were 10–15 posts in length.^18^ It may be that the inherent demand for brevity in each post forces the author to be specific and precise in their information delivery—ultimately streamlining the presentation of content—and thus longer tweetorials are still engaging while offering a more in-depth analysis.^2^

While the focus on a single social media platform inherently limits the generalizability of these findings, our results offer some insights into features associated with reach of education posts. Content that is visually appealing, creative, and about a topic that many users in that social media community would find practically useful is the most likely to be widely shared. These findings are somewhat intuitive because they mirror effective teaching in other formats such as lectures,^17^ bedside teaching,^19^ and flipped-classroom paradigms.^20^ Many of these elements were those empirically suggested by Tony Breu^2^ (often credited as the first physician to popularize the tweetorial within the medical community) in one of the first published guides on authoring tweetorials.

Notably, of the features that were unmodifiable, being a non-neurologist was strongly correlated with a high XF. This is likely driven by the fact that authors from larger subspecialties (such as emergency medicine and internal medicine, who did write neurology-themed tweetorials) can accrue larger following within their larger specialty. While the GEE can account for differences in each author, the collective effect was not mitigated by the GEE and was captured by the quasi-likelihood under the independence model criterion as an important variable. Said differently, although followership could be adjusted for, a large positive deviation in followers does affect the XF for the author's tweetorial.

We also found several controllable elements that had a negative impact on sharing. These include being CME accredited, the presence of polls, and a high score for positive tone in the language analysis. Although the fact that CME accreditation resulted in less impact could suggest that X users may gravitate to unverified sources, a significant number of CME-accredited posts were posted by specific CME accounts. These accounts recruit experts in the field to be authors, but the authors themselves do not post the threads. In this way, the CME accounts are different from most other X accounts in the data set, which were physician’s or trainee's personal accounts. We hypothesize that the low impact of CME-accredited tweetorials more likely reflects low engagement with institutional or commercial accounts and that the development of a personal teaching “brand” is important for educators on social media platforms, although this deserves further study.

While polls and audience response systems are an evidenced-based technique to engage learners in the classroom,^21^ tweetorials with polls were associated with less sharing. This may reflect what is known about student preferences—students consistently learn more from methods of teaching that demand active learning; however, they prefer and more highly rate passive learning.^22^ In the social media space, where all learning is optional, users may shy away from threads that require active participation.

While a high score for positive tone in the language analysis predicted less sharing, the effect was small. This contradicts what has been seen in another study of health care professionals in which supportive tweets predicted a higher number of retweets among cardiologists on X.^23^ Conversely, a high score on negative tones had no impact on sharing. Thus, the lower impact of positive-toned first posts cannot be interpreted as that negative-toned or more “scandalous” posts gain more attention, although this may be true outside #NeuroTwitter.^24^ Thus, while users may want to limit being overly positive, there was no other feature of language evaluation that positively correlated with sharing.

Despite the abovementioned findings being informative for authors who want to post education content in social media, our study has several limitations that affect interpretation and application of our data. Regarding content sampling and data set creation, a major limitation is the content on X is not well archived. Our research highlights how difficult it can be to capture an unbiased sample. There are many teaching threads written on X that do not explicitly state “tweetorial” AND “neuro/neurology” and thus would be missed by our initial review of X. Despite our attempt to curate all neurology-themed tweetorials, we could most reliably capture teaching threads when the author grouped all their tweetorials in “Moments,” a feature that has since been disabled by X. As such, our data set is enriched in these threads (147/392, 37%) and thus likely represents a sampling bias weighed toward the group of sophisticated X users who have authored multiple tweetorials. Indeed, 16% of the user handles (14/88) had authored more than 5 tweetorials each and accounted for 44% of all tweetorials (175/392). It was impossible to capture the exact number of followers an author had when they posted their tweetorial. Although we accounted for the author effect using the GEE, it is likely that residual confounding still exists and that the authors characteristics, such as number of followers and number of previously authored tweetorials, may account for an unmeasurable effect. The impact of authorship was highlighted in that non-neurologists have, on average, higher XF tweetorials likely because, on average, authors from larger specialties can amass larger following. This collective between-group difference was not mitigated by the GEE. Similarly, our data set was enriched in vascular and neurocritical care topics; what makes these tweetorials widely shared may be different from that for other subspecialties in neurology.

Additional limitations include the interrater reliability and the limitations in language assessment. There was moderate (K = 0.45 and K = 0.58) agreement for what is a “General Neurology Topic” and “Clearly Stated Topic,” respectively, and substantial agreement (K = 0.67) for the style of the first post. However, small variations may have influenced the impact of their subjective measures. In the language analysis, we chose to only include the language of the first post because this is the post that is most likely to determine the social media user's interest in the topic, but we may have found a different effect from including the language from the entire tweetorial for analysis. Finally, this represents an analysis from 1 site, and results may not be generalizable to other social media platforms.

Overall, this study reflects a fundamental challenge in education research: the process of education is much easier to study than the ultimate desired effect of medical education, which is improved clinical care by the learner. While our work highlights how authors can produce content that is most likely to be widely liked and shared, future work should examine the next step in the online education pathway, specifically how to assess the impact on learners who have been reached by online content.

## Appendix Authors

**Table:**

Name	Location	Contribution
Catherine S.W. Albin, MD	Department of Neurology, Emory University School of Medicine	Drafting/revision of the manuscript for content, including medical writing for content; major role in the acquisition of data; study concept or design; analysis or interpretation of data
Tianwen Ma, PhD	Division of Biostatistics, Rollins School of Public Health	Analysis or interpretation of data
Gabriela F. Pucci, MD	Department of Neurology, University of Pittsburgh	Drafting/revision of the manuscript for content, including medical writing for content; major role in the acquisition of data
Aaron S. Zelikovich, MD	Department of Neurology, Weill Cornell Medical College	Drafting/revision of the manuscript for content, including medical writing for content; major role in the acquisition of data
Eric C. Lawson, MD	Department of Neurology, Emory University School of Medicine	Drafting/revision of the manuscript for content, including medical writing for content; major role in the acquisition of data
Neil Dhruva, MD	Emory University School of Medicine	Major role in the acquisition of data
Simone Masiero	Web Developer	Major role in the acquisition of data
Aarti Sarwal, MD	Department of Neurology, Wake Forest School of Medicine	Drafting/revision of the manuscript for content, including medical writing for content; study concept or design
Neha S. Dangayach, MD, MSCR	Departments of Neurology and Neurosurgery, Icahn School of Medicine at Mount Sinai	Drafting/revision of the manuscript for content, including medical writing for content; study concept or design
Aaron L. Berkowitz, MD, PhD	Department of Neurology, University of California, San Francisco	Drafting/revision of the manuscript for content, including medical writing for content; major role in the acquisition of data; study concept or design
Nicholas A. Morris, MD	Department of Neurology, University of Maryland School of Medicine	Drafting/revision of the manuscript for content, including medical writing for content; study concept or design
Lyell K. Jones, Jr.	Department of Neurology, Mayo Clinic	Drafting/revision of the manuscript for content, including medical writing for content; major role in the acquisition of data; study concept or design; analysis or interpretation of data

## Glossary

CME: continuing medical education
FND: functional neurologic disorder
GEE: general estimating equation
GIF: graphics interchange format
IQR: interquartile range
XF: X Factor
